# Diagnostic accuracy of using capnography in verification of nasogastric tube placement among adult patients in hospital settings: Protocol of a diagnostic study

**DOI:** 10.1371/journal.pone.0292667

**Published:** 2023-10-23

**Authors:** Janita Pak Chun Chau, Danny Wah Kun Tong, Suzanne Hoi Shan Lo, Savina Yee Man Sze, Molly Lai Mei Kwok, Peter Chi Keung Lai, Harris Kai Cheong Lam, Josephine Yuen Man Chung, Xu Liu, Wai Tong Chien, Kai Chow Choi

**Affiliations:** 1 Nethersole School of Nursing, The Chinese University of Hong Kong, Hong Kong, Hong Kong; 2 Nursing Services Department, Hospital Authority Hong Kong, Hong Kong, Hong Kong; 3 Shatin Hospital, Hospital Authority Hong Kong, Hong Kong, Hong Kong; 4 Queen Mary Hospital, Hospital Authority Hong Kong, Hong Kong, Hong Kong; 5 Ruttonjee and Tang Shiu Kin Hospitals, Hospital Authority Hong Kong, Hong Kong, Hong Kong; 6 Prince of Wales Hospital, Hospital Authority Hong Kong, Hong Kong, Hong Kong; Aristotle University of Thessaloniki School of Veterinary Medicine, GREECE

## Abstract

**Objective:**

To determine the diagnostic accuracy of end-tidal carbon dioxide (ETCO_2_) detection using capnography for verifying the correct placement of nasogastric tubes (NGTs) among adult patients in hospital settings.

**Materials and methods:**

A prospective observational diagnostic study will be conducted. Patients ≥ 18-year-old and requiring the insertion of an NGT will be recruited using a convenience sampling method from 39 general medical and geriatric wards, intensive care units, accident and emergency departments, and subacute/rehabilitation/infirmary wards in 21 acute or subacute/convalescent/extended care hospitals. ETCO_2_ detection by sidestream capnography, which indicates an airway intubation of an NGT when a capnogram waveform or an ETCO_2_ level > 10mmHg (1.33 kPa) occurs, will serve as the index test. The reference standards will be the X-ray performed and pH value of gastric aspiration (pH ≤ 5.5) after the index test. Each participant will be included only once. Sensitivity, specificity, positive predictive value, negative predictive value, and area under the receiver operating characteristic curve of capnography will be calculated to assess the diagnostic performance of capnography. The variability in diagnostic accuracy in participants with different characteristics will be explored by using chi-squared or Fisher’s exact tests. The time spent and the cost of the tests will be compared using the paired t-test. All statistical tests will be two-sided with a level of significance set at 0.05.

**Discussion:**

This study will provide evidence on the diagnostic accuracy of capnography in verifying NGT placement and its applicability to patients in hospitals settings, since this evidence is limited in the current literature. In addition, it will help identify the optimal combination of tests in verifying the correct placement of NGTs and inform the update of clinical practice guidelines and stakeholders’ decisions on the adoption of ETCO_2_ detection as a routine method for verifying NGT placement.

**Trial registration:**

ClinicalTrials.gov ID: NCT05817864.

## Introduction

Nasogastric tube (NGT) placement is a common practice in hospital settings for assessment, nutrition support, and administration of medication [1,2]. Unfortunately, misplacement of NGTs is common, with an estimated 60% of misplacements resulting in detrimental effects [3,4]. In fact, in over 80% of these misplacements the NGTs were placed in the respiratory tract, leading to serious pulmonary complications, including aspiration pneumonia or respiratory failure [4,5]. Chest/abdominal X-ray is the gold standard for verifying correct NGT placement in the stomach. However, radiography is not always available conveniently at the bedside [6]. Other methods for verifying NGT placement include biochemical measurements of aspirates for pH value or presence of bilirubin, trypsin or pepsin, spring gauge pressure manometer, magnetic detection, ultrasonography, auscultation, and visual inspection of aspirates [7–11]. Factors often compromised the accuracy of these methods include the inability to aspirate adequate fluids for testing, difficulty in visually differentiating the colour change in test strips, use of antacids, reflux of gastric contents, or patients with altered level of consciousness [7,8,11]. Therefore, a reliable and effective bedside test to verify NGT placement is required.

An increasing amount of evidence suggests that end-tidal carbon dioxide (ETCO_2_) detection may be a promising method to verify NGT placement [2,6]. Capnography comprises the continuous analysis and recording of CO_2_ in respiratory gases. The placement of the NGT within the airway is defined as the detection of ETCO_2_ levels exceeding 15 mmHg (2 kPa) [12–15]. An NGT is also considered to be placed in the respiratory tract if a change from purple to yellow is detected by a colorimetric ETCO_2_ device [14]. We conducted a meta-analysis of nine studies involving 651 NGT placements and found that the pooled results for sensitivity and specificity of ETCO_2_ detection in differentiating respiratory and gastrointestinal (GI) placement of NGTs among critically ill adult patients were high, both of which were 0.99 [16]. After a comprehensive search of databases from 2009 to March 2021, the review was updated, and seven new studies were identified. Of the 16 included studies with 1,878 NGT placements, the pooled results for sensitivity and specificity were 0.96 and 0.99, respectively [17]. The results consistently suggest that capnography and colorimetric capnometry may be of comparable diagnostic accuracy to radiography in their ability to differentiate between respiratory and GI tube placement in critically ill adult patients. However, the limited number of studies conducted in general wards and other hospital settings renders the determination of diagnostic accuracy in this patient population uncertain [13,18].

To determine the applicability of ETCO_2_ detection in clinical settings, Cheng and Lee evaluated the diagnostic accuracy of colorimetric capnometry in verification of feeding tube placement in 26 patients (with 71 doubtful NGT placements) recruited from three medical extended wards in a public hospital in Hong Kong [13]. The specificity of the colorimetric capnometry was 98.6%, while the sensitivity could not be determined. A team of community nurses conducted a pilot study between March and April 2021 to examine the use of capnography in verifying correct NGT placement. Two devices were used, namely EMMA Capnograph and Rad-97 Pulse Co-oximeter. A total of 12 older adults (mean age 80.3 years, range 55–89 years old) residing in old age homes were recruited. Nine of them were receiving lansoprazole, and one was receiving famotidine. Of the 15 episodes of NGT insertion performed among the 12 older adults (seven used EMMA Capnograph and eight used Rad-97), none of the episodes detected ETCO_2_ and crisp waveforms after testing of tube placement in the stomach by the Whoosh test [13].

Despite the potential benefits, some gaps regarding the use of ETCO_2_ detection were identified in the literature. First, the majority of included studies in the two meta-analyses examined critically ill patients or patients with mechanical ventilation [16,17]. Also, the two studies in Hong Kong were conducted among a small sample of hospital patients and community-dwelling older adults. Thus, there is limited evidence on the diagnostic accuracy of ETCO_2_ detection in acute and rehabilitation settings, particularly general wards, accident and emergency departments (AEDs) and intensive care units (ICUs). Second, the sensitivity and specificity of ETCO_2_ detection shown in the meta-analyses were less than 100%. Factors such as recent ingestion of carbonated fluids or poor connection between NGTs and ETCO_2_ detectors may result in false positive or false negative results [16,17,19]. Therefore, further examination of strategies to enhance the diagnostic accuracy of ETCO_2_ detection is needed. A protocol on the procedure of carrying out ETCO_2_ detection is also needed to avoid the possibility of false results caused by mal operation of the device or tubing misconnections. Third, ETCO_2_ detection is suggested to be effective only in differentiating between respiratory and GI placement of the NGTs. The use of this method to verify correct tube placement in the stomach, either alone or in combination with other index tests or reference standards, is yet to be determined. Thus, this study aims to discern the diagnostic accuracy of ETCO_2_ detection using capnography in verifying the correct placement of NGTs among adult patients in hospital settings.

## Materials and methods

### Study design

A prospective observational diagnostic study will be conducted to assess the sensitivity and specificity of capnography in detecting the correct placement of NGTs using the reference standards of radiography and measurement of aspirates for pH value. This study was registered with ClinicalTrials.gov (ID: NCT05817864). We reported this study protocol following the recommendations of the STARD (Standards for Reporting Diagnostic Accuracy) statement [20] and the SPIRIT (Standard Protocol Items: Recommendations for Interventional Trials) 2013 statement [21].

### Participants

Individuals who meet the following inclusion criteria will be recruited: (1) 18 years old or above; (2) admitted to the general medical and geriatric wards, ICUs, or subacute/rehabilitation/infirmary wards in subacute/convalescent/extended care hospitals or visiting AEDs in acute hospitals; and (3) requiring the insertion of an NGT into the stomach for assessment and monitoring, nutritional support, drainage, and medication administration during the study period. Individuals will be excluded from the study if they are receiving a life-saving intervention at the time of recruitment.

A convenience sampling method will be adopted in this study. The participants will be recruited in 39 wards/units across 21 acute or subacute/convalescent/extended care hospitals. Each participant will be included in the study once.

### Test methods

#### Index test

Capnography, which monitors ETCO_2_, the percentage concentration, or partial pressure of CO_2_ at the end of exhalation, will serve as the index test. It will be performed by connecting the end of the NGT with the sensor of the bedside sidestream capnography device (Medtronic Capnostream 35 Portable Respiratory Monitor) through an adapter [22]. After connecting the NGT to the sensor, the ETCO_2_ level should be read after a minimum of six breaths or one minute to avoid confusion resulting from CO_2_ in the stomach [13,18,23,24]. Placement within the airway is defined as detecting a capnogram waveform or an ETCO_2_ level > 15 mmHg (2.0 kPa) [15].

#### Reference standard

The reference standard is the test, series of tests, or set of procedures used to determine the presence or absence of the target condition in individuals, which in this study is airway intubation of an NGT [25]. This study will use radiography (chest/abdominal X-ray) as the reference standard for determining the correct placement of NGTs. Radiology will be performed as soon as possible and interpreted by a physician. A repeat radiography will be performed if necessary. We will also use pH-Fix-4.5–10 to determine the pH value, and its accuracy is +/- 0.2 with minimum increments of 0.5. A pH of 5.5 or below indicates gastric placement [2].

### Procedures

The principal investigators and co-investigators will provide two 1.5-hour training sessions to the designated nurses (DNs) at each study site, one e-learning session and one real-time synchronised online session. A DN is a registered nurse, advanced practice nurse, associate nurse consultant, or nurse consultant working in the study site. The training will cover principles, types, and clinical applications of capnography, representative capnographic waveform, limitations and issues of concern regarding capnography, the study protocol, procedures for ETCO_2_ detection using the bedside capnography device, and study documentation. All DNs must attend both training sessions and pass a 15-item quiz to demonstrate their knowledge of capnography monitoring. To ensure adherence to the study protocol, the project team will conduct non-participant observation once for each DN. The DN of each study site will assess participants’ eligibility based on the eligibility criteria, and no pre-screening by ward nurses will be performed to avoid selection bias.

**Fig 1:**
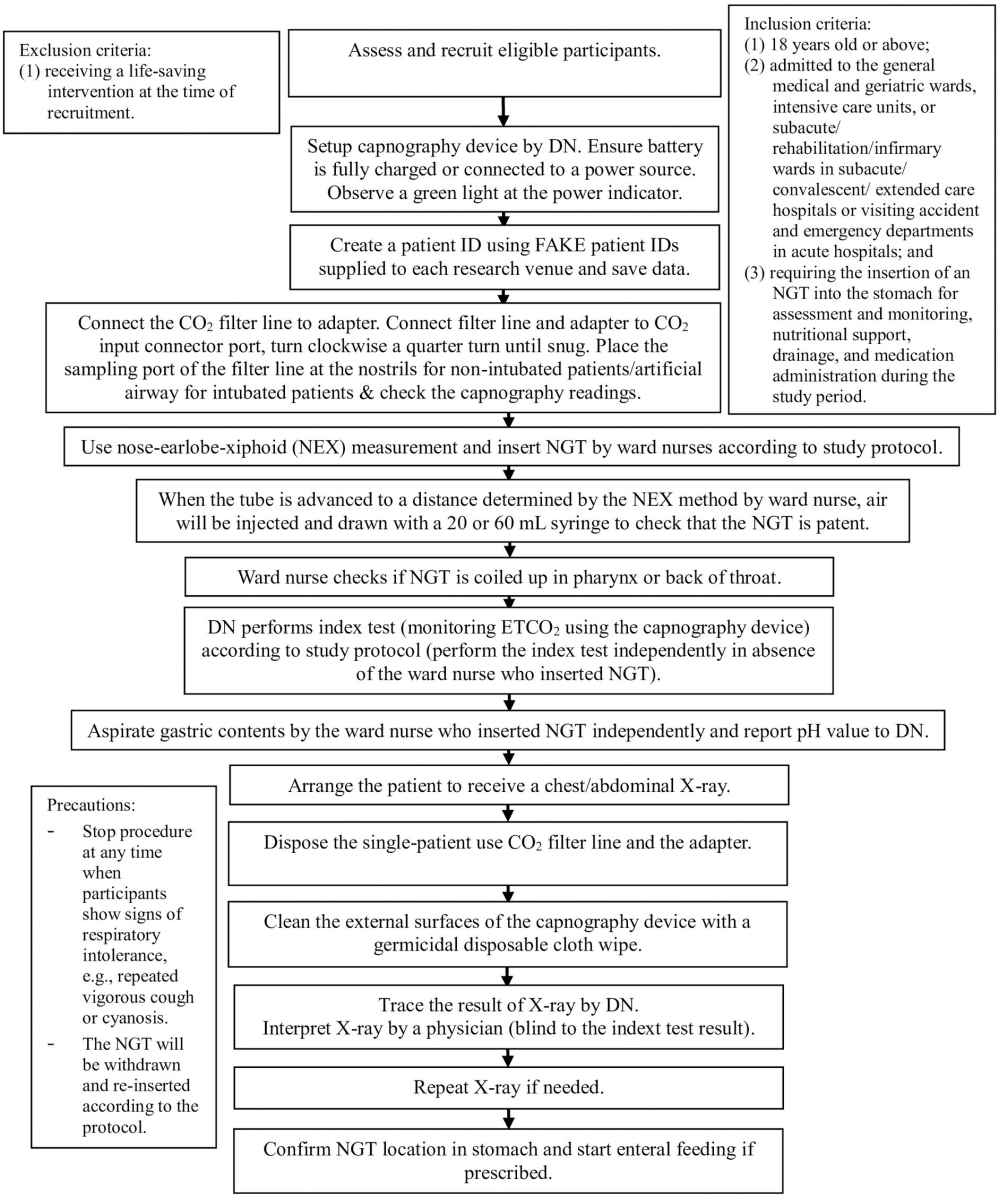
The flow of participants and data collection procedures in the study. This is the Fig 1 legend. (Abbreviation: DNs: Designated nurse(s) of the study venues; ETCO_2_: End-tidal carbon dioxide; ID: Identity; NGT: Nasogastric tube).

Fig 1 depicts the flow of the participants and data collection procedures. Before insertion, the DN will set up the capnography device according to the manufacturer’s instructions, connect the Microstream Advance Adult-Pediatric Intubated CO_2_ filter line to the adapter, and then connect it to the CO_2_ input connector port on the monitor. The DN will check proper device function by placing the sampling port of the filter line at the nostrils for non-intubated patients or artificial airway for intubated patients and checking the capnography readings (i.e., observing the CO_2_ waveform and obtaining the ETCO_2_ reading).

Ward nurses in the study site responsible for insertion of the NGT will estimate the distance to placement using the nose-earlobe-xiphoid (NEX) measurement and then insert the NGT according to the standard clinical practice guideline of the Hospital Authority Hong Kong (without connecting the capnography device at this point). The NGT used in all cases will be either a large bore NGT or a fine bore NGT. When the tube is advanced to a distance determined by the NEX method, ward nurses will inject and draw air with a 20 or 60 mL irrigation syringe to ensure tube patency and check if the NGT is not coiled inside the oral cavity.

Afterward, the DN will perform the index test (monitoring the ETCO_2_ using the capnography device) independently in the absence of the nurse who inserted the NGT in the following manner: i) Connect a new 20 or 60 mL syringe to the NGT and flush in 10 mL of air to clear any blockage; ii) Remove the syringe, connect the capnography device to the distal end of the NGT (by firmly connecting the device, the filter line with adapter and the NGT); iii) Check for any capnographic waveform and ETCO_2_ reading after a minimum of six breaths or one minute, whichever is longer, and record the results. To minimise bias in the study, the DN will not disclose the results of ETCO_2_ detection to the related ward nurses, participants, radiographer, or the physicians/radiologists who will interpret the X-ray imaging.

The ward nurses will independently aspirate gastric contents for pH measurement and report the pH value to the DN for record. The procedure will be stopped immediately if participants show signs of respiratory intolerance, such as repeated vigorous cough or cyanosis [26]. Then the patient will be arranged to receive a chest/abdominal X-ray. The DN will trace the X-ray results, which will then be interpreted by a physician (blind to the result of the index test) to confirm the position of the NGT. Enteral feeding (if prescribed) will begin once the position of the NGT is confirmed, otherwise, the NGT will be withdrawn and re-inserted according to the protocol.

### Data collection

The DN will record the result of ETCO_2_ detection (i.e., presence of any capnographic waveform and ETCO_2_ value and the number of index tests performed per participant) and follow up with the X-ray and the pH test results; and location of NGT (stomach, esophagus, or tracheobronchial tree). These results will be recorded on the data collection form.

The DN will also collect participants’ demographic and clinical data (S1 File), including age, gender, setting (ward/unit and type of hospitals), principal medical diagnosis, reasons for current AED visit or hospitalisation, past medical and surgical history, level of consciousness (assess using Glasgow Coma Scale), level of fitness or frailty (assess using Clinical Frailty Scale), respiration status and support, indication for NGT insertion, type of NGT, mode, amount and frequency of enteral feeding, type of diet or enteral formula, fluid and food intake within 6 hours before NGT insertion, medications administered within 12 hours of NGT insertion, results and duration of the index test and reference test/s performed, complications or adverse events associated with insertion and the testing (index test and reference test/s), cost of the index test, and cost of the reference test/s used.

### Study duration

The duration of the research will be two years, and data collection will begin in July 2023.

## Data analysis

The sample size is determined to give the study adequate precision to estimate the sensitivity and specificity of using capnography in detecting the placement of NGTs using the reference standards of measurement of radiography and aspirates for pH value. In anticipation of sensitivity and specificity of values greater than 90% [17], a sample size of at least 500 participants would be sufficient to estimate these parameters with a margin of error of at most ±3% at a significance level of 0.05. The sample size calculation was performed using PASS 16 (NCSS, Kaysville, USA). Eligible participants will be evenly recruited from the 39 participating wards/units. The target sample size will therefore be rounded up to 507 with 13 eligible participants per each of the 39 wards/units.

IBM SPSS 26.0 (IBM Crop., Armonk, NY) will be used to conduct the data analysis. Participants’ demographic and clinical characteristics will be presented by appropriate descriptive statistics, including mean ± standard deviation, median (inter-quartile range), and percentages for normally distributed, skewed continuous, and categorical variables respectively. Any cases with missing data on the index test or the reference standard results will be excluded from the test accuracy analysis. The true positives (i.e., NGT placements that are tested positive by both index test and reference test/s), false negatives (i.e., NGT placements that are tested negative by index test but positive by reference test/s), true negatives (i.e., NGT placements that are tested negative by both index test and reference test/s), and false positives (i.e., NGT placements that are tested positive by index test but negative by reference test/s) will be calculated, based on which the sensitivity, specificity, positive predictive value, and negative predictive value of the capnography test will be calculated with reference to a combination of X-ray and aspirate pH test using the following formulas:

$$\text{Sensitivity}:\;\text{True}\;\text{Positive}/\left(\text{True}\;\text{Positive}+\text{False}\;\text{Negative}\right)\times 100\%$$

$$\text{Specificity}:\text{True}\;\text{Negative}/\left(\text{True}\;\text{Negative}+\text{False}\;\text{Positive}\right)\times 100\%$$

$$\text{Positive}\;\text{Predictive}\;\text{Value}:\text{True}\;\text{Positive}/\left(\text{True}\;\text{Positive}+\text{False}\;\text{Positive}\right)\times 100\%$$

$$\text{Negative}\;\text{Predictive}\;\text{Value}:\text{True}\;\text{Negative}/\left(\text{True}\;\text{Negative}+\text{False}\;\text{Negative}\right)\times 100\%.$$

The Area Under the Receiver Operating Characteristic Curve (AUC) of capnography will be calculated to assess the overall diagnostic performance of capnography. The test accuracy level is considered high when the AUC value is ≥ 0.9 [25].

The variability in diagnostic accuracy in participants from different age groups, gender, level of consciousness, level of frailty, type of illness, respiration status and support, drug use, fluid or food intake, types of hospitals and units will be explored by using chi-squared or Fisher’s exact tests, as appropriate. The time spent and the cost of the index test (capnography monitoring) and the reference tests (radiography and aspirate pH test) will be compared using the paired t-test. All statistical tests are 2-sided with level of significance set at 0.05. All cost data will be expressed in Hong Kong and US dollars and the procedural time will be expressed in hours and minutes.

### Pilot study

To understand the feasibility of the methods used in this study, we will conduct a pilot study before committing to the fidelity required for this multicentre study. We will select three cases in each in-patient setting, AED, and ICU. The ward nurses and DNs will perform NGT insertions and tests strictly according to the prespecified procedures. Besides participants’ demographic and clinical data and the results of the index test and reference tests specified in the data collection form, quantitative process measures and qualitative reviews will be used to establish the feasibility of the study.

The following process will be evaluated: DNs’ and ward nurses’ adherence rate to the protocol, total time used (from capnography calibration to receiving the results of the reference tests), and cost of the index test and the reference tests. The experiences of participating in the test of the DNs and ward nurses will be collected through semi-structured interviews with questions on the performance and completeness of the procedures and tests, feasibility of the procedures and tests, and usability of the capnography, pH test, and X-ray.

### Ethical considerations

The research team will protect participants’ rights and safety by adhering to local laws, Declaration of Helsinki, institutional policies, and ICH-GCP. There will be an emphasis on voluntary participation, and participants will be informed they can withdraw at any time from the study without consequences. Agreement will be made in advance with personnel in charge of the study venues for arranging participant recruitment. Before the study, participants will be informed of study details and asked to sign an informed consent form (See S2 File, information sheet and consent form for patients). If a potential participant lacks capacity to consent due to an acute illness or medical conditions that impairs cognition, the patient’s next of kin will be contacted, provided with a thorough explanation of the potential risks, benefits, and procedures of the study and will be asked to sign an informed consent form. The reasons why the patient is unable to provide consent (e.g., patient is non-responsive due to a medical or surgical condition or medication administered to treat the conditions) will be documented in the research file/chart and reviewed by the principal investigator and co-investigators. The patient and patient’s next of kin may withdraw from the study at any point, and their informed refusal will result in immediate data deletion.

All data collected will be kept confidential and used solely for this study. After data collection, the DNs of each study venue will store the raw data in a password-protected file and send it to the lead principal investigator (PI). Other researchers in the research team who want to use the data will need to be authorised by the lead PI and sign a data-sharing agreement. All electronic documents will be encrypted and stored in password-protected files, and all paper documents will be locked securely.

All identity information including the names and identity card number of the patients will not be collected. Each participant will be assigned an identity number. The data will also be analysed anonymously. All DNs will be trained and reminded on the importance of confidentiality and safe keeping of the data. They will be asked to sign a confidentiality pledge which highlights the importance of using appropriate safeguards to protect the confidentiality of the information contained within the data set. No attempt will be made to contact individuals or family members of individuals whose information is contained in the data set.

This study has received ethical approval from The Joint Chinese University of Hong Kong–New Territories East Cluster Clinical Research Ethics Committee (CREC Ref. No.: 2022.073), Hospital Authority Central Institutional Review Board (Ref. No.: CIRB-2023-030-4), and Institutional Review Board of the University of Hong Kong/Hospital Authority Hong Kong West Cluster (IRB Ref. No.: UW23-332). Any amendments to the protocol will be sought from the research ethics committees.

## Discussion

This study is aimed at determining the diagnostic accuracy of ETCO_2_ detection using capnography in verifying the correct placement of NGTs among adult patients in hospital settings. Ensuring the correct placement of NGTs is a necessary and important step to ensure patient safety. Despite the various methods examined in literature for verifying NGT placement, the diagnostic accuracy of these methods is still suboptimal and often compromised by different patient- or equipment-related factors such as availability of aspirates or conditions of the test devices [7,16,17]. This study will provide evidence on the diagnostic accuracy of capnography in verifying NGT placement, and its applicability to patients in hospitals settings, evidence of which is limited in the current literature.

In this study, capnography will be examined in combination with the reference standards, including radiography and testing for pH values in gastric aspirates. This examination approach will identify the optimal combination of test results in verifying the correct placement of NGTs. A multicenter, observational trial of 85 patients undergoing general anaesthesia, orotracheal intubation and mechanical ventilation has suggested using a ETCO_2_ cutoff value of 25.5 mmHg for NGT tracheal misplacement and a pH cutoff value of 4.25 for NGT correct gastric placement for achieving a higher level of accuracy in determining gastric placement of NGTs [27]. Indeed, given that the use of ETCO_2_ may be limited in merely differentiating between tracheal and GI placement of NGTs, the use of more than one index test could be superior in increasing the diagnostic accuracy. This study will help determine a fast and effective method for detecting NGT placement in the respiratory tract, which is crucial to preventing respiratory complications associated with misplaced NGTs. Furthermore, an algorithm illustrating the decision making based on the results of these tests can be developed to guide practice. The results of this study will also inform the update of clinical practice guidelines regarding the recommendations across the Hospital Authority Hong Kong in verifying NGT placements in acute and rehabilitative settings.

Building on the results of this study, further studies can be initiated to examine the cost-effectiveness of ETCO_2_ detection. Qualitative evaluation of healthcare professionals’ feedback on using this method and exploration of patient experiences would be necessary to better understand the applicability of the method. More studies designed to test this method’s diagnostic accuracy in verifying tube location before each use, and among people with continuous feeding, would also be worthwhile. The results will provide valuable evidence in informing stakeholders’ decisions on the adoption of ETCO_2_ detection as a routine method for verifying NGT placement.

## Supporting information

S1 File Data collection form. This is the S1 File legend.
(DOCX)

S2 File SPIRIT reporting checklist. This is the S2 File legend.
(DOCX)
