# Supplementary material for: Diagnostic accuracy of using capnography in verification of nasogastric tube placement among adult patients in hospital settings: Protocol of a diagnostic study
Source: PLoS One. 2023 Oct 23;18(10):e0292667. doi: 10.1371/journal.pone.0292667 (PMC10593214; doi:10.1371/journal.pone.0292667)
Supplement: S1 File — This is the S1 File legend. (DOCX) [file pone.0292667.s001.docx]

**S2: Data Collection Form**

**Demographic and clinical information sheet for participants**

| **Case code** |  |
| --- | --- |

1. **Demographic information**

| **Recruitment date:** | |  | **Informed consent is obtained?** | **🞎** Yes | **🞎** No |
| --- | --- | --- | --- | --- | --- |
| **Test date & time:** | | Date (DD/MM/YYYY): | Index Test Assessment: Start Time, 24-hr time format (HH:MM): | | |
|  | **Study venue:**  **Cluster: ______________________________**  **Hospital: _____________________________**  **Department: __________________________**  **Ward: _______________________________** | | | | |

**Age:** _______ DOB (DD/MM/YYYY): __________________

**Gender: 🞎** M 🞎 F

**Date and time of hospital admission (**DD/MM/YYYY; 24-hr time format HH:MM): __________________ ; __________

**Date and time of ICU admission (if applicable) (**DD/MM/YYYY; 24-hr time format HH:MM): __________________ ; __________

1. **Clinical information**

1. Principal diagnosis & ICD-9-CM code/s

| **_______________________________________________________________**  **_________________________________________________________________________________** |
| --- |

2. Reasons for current A&E visit or hospitalisation (can list more than one reason)

| **________________________________________________________________**  **________________________________________________________________** |
| --- |

3. Applicable to A&E Department

Transfer from nursing homes/residential care facilities

**🞎** Yes **🞎** No

4. Applicable to ICU

*4.1 Respiration status and support:*

4.1.1 Unassisted spontaneous breathing **🞎** Yes **🞎**No

4.1.2 Non-invasive ventilation support **🞎** Yes **🞎**No

4.1.3 Presence of artificial airway: **🞎** Yes **🞎**No

4.1.3.1 Endotracheal tube (ETT) size: _____mm

4.1.3.2 Characteristics of the endotracheal tube (tick all appropriate boxes)

- Cuff: **🞎** Cuffed tube, cuff inflated **🞎** Cuffed tube, cuff deflated **🞎** Non-cuffed tube
- Type: **🞎** Nasal ETT **🞎** Oral ETT **🞎** Double-lumen ETT

4.1.3.3 Tracheostomy tube size: ______ mm

4.1.3.4 Characteristics of the tracheostomy tube (tick the appropriate boxes)

- Cuff: **🞎** Cuffed tube, cuff inflated **🞎** Cuffed tube, cuff deflated **🞎** Non-cuffed tube
- Type: **🞎** Single-cannula tube **🞎** Double-cannula tube#
- For Double Cannula Tube#, state the type of inner tube used:

**🞎** fenestrated inner tube **🞎** non-fenestrated inner tube

*4.2 Ventilation mode: _______________*

4.2.1 Mode: **🞎** PC **🞎** VC **🞎** SIMV+PS **🞎** PS **🞎** Others (please specify): __________

4.2.2 Fraction of Inspired Oxygen (FiO2): ____

4.2.3 Positive End Expiratory Pressure (PEEP): ____cmH2O

5. Level of consciousness (Glasgow Coma Scale)

GCS Component Score:

E___ V ___ M ___

GCS Total Score: _________

**Reference:** Glasgowcomascale.org (2015). What is GCS - Glasgow Coma Scale. [online] Available at: <http://www.glasgowcomascale.org/what-is-gcs/

6. Medical and health condition

*6.1 Clinical Frailty Scale*

**Assess CFS score according to patient’s ability TWO weeks before A&E attendance or hospital admission.**

Note: Ask the patient or their carer/next of kin/care home staff what the patient’s capability was TWO weeks ago.

CFS Score: _________

**Reference**: Rockwood K, & Theou O. (2020). Using the Clinical Frailty Scale in allocating scarce health care resources. *Canadian Geriatrics Journal, 23*(3), 210-215.

*6.2 Past medical and surgical history*

| **________________________________________________________________________________________________________________________________________________________________________________________________________________________________________________________** |
| --- |

7. Indication for NGT

| 🞎 Assessment & monitoring  🞎 Medication  🞎 Drainage  🞎 Nutrition support  Indication/s for nutritional support (you can choose more than one option):  🞎 Dysphagia  🞎 Infections  🞎 Respiratory failure  🞎 Stroke  🞎 Advanced cognitive impairment  🞎 Motor neurone disease  🞎 Parkinson’s disease  🞎 Pressure injury  🞎 Preoperative  🞎 Postoperative (drainage)  🞎 Others: please specify ______________________________________________________  🞎 Others: please specify |
| --- |

8. Type of NGT

| 🞎 Size of NGT, Fr_______  (Polyvinyl chloride/ polyurethane/ silicone/ Others: ______________) (circle as appropriate) |
| --- |

9. Special order besides tube feeding

🞎 No

🞎 Yes 🞎 Nil by mouth (NPO) since when (Date: __________________ Time: ______________)

🞎 NPO except medication

🞎 Oral feeding + Tube feeding

🞎 Others: (please specify) __________________________________________________

10. Enteral feeding information

| **10.1 Mode:** | 🞎 Intermittent  🞎 Continuous  🞎 NPO except medication |
| --- | --- |
| **10.2 Milk formula**  **+/- diet prescribed:** | __________________________________________ |
| **10.3 Prescribed amount:**  **(e.g., XX mL QXH)** | ______________________________________________________ |

11. Intake before NGT insertion:

*11.1 Fluid intake within 6 hours before NGT insertion*

| **Time** | **Intake** | **Amount (in mL)** |
| --- | --- | --- |
|  | 🞎 water  🞎 enteral formula (type: )  🞎 other liquid (type: ) |  |
|  | 🞎 water  🞎 enteral formula (type: )  🞎 other liquid (type: ) |  |
|  | 🞎 water  🞎 enteral formula (type: )  🞎 other liquid (type: ) |  |
|  | 🞎 water  🞎 enteral formula (type: )  🞎 other liquid (type: ) |  |
|  | 🞎 water  🞎 enteral formula (type: )  🞎 other liquid (type: ) |  |
|  | 🞎 water  🞎 enteral formula (type: )  🞎 other liquid (type: ) |  |

***11.2 Food intake within 6 hours before NGT insertion***

| **Time** | **Intake** | **Amount** |
| --- | --- | --- |
|  |  |  |
|  |  |  |
|  |  |  |

*11.3 Medications (oral) within 12 hours prior to NGT insertion*

|  | **Name of Medication** | **Dosage** | **Route** | **Frequency** | **Administration time of the last dose** |
| --- | --- | --- | --- | --- | --- |
| **1** |  |  |  |  |  |
| **2** |  |  |  |  |  |
| **3** |  |  |  |  |  |
| **4** |  |  |  |  |  |
| **5** |  |  |  |  |  |
| **6** |  |  |  |  |  |

*11.4 Medications (intravenous/injection) within 12 hours prior to NGT insertion:*

|  | **Name of Medication** | **Dosage** | **Route** | **Frequency** | **Administration time** |
| --- | --- | --- | --- | --- | --- |
| **1** |  |  |  |  |  |
| **2** |  |  |  |  |  |
| **3** |  |  |  |  |  |
| **4** |  |  |  |  |  |
| **5** |  |  |  |  |  |
| **6** |  |  |  |  |  |

12. Test information:

| *12.1*  *Index test details*  Connect the capnography device for a duration of one minute.  After one minute, record the ETCO_2_ reading and waveform. | 🞎 Verify that the capnography device is operating properly.  Waveform Seen 🞎 Yes 🞎 No | | 🞎 The ETCO_2_ range (within 1 minute after a minimum of six breaths or one minute)* ( ____ mmHg/kPa) |
| --- | --- | --- | --- |
|  | Remarks: | | |
| *12.2 Reference test(s) used* | 🞎 Gastric aspirate (pH test)  🞎 X-ray | | |
|  | Remarks: | | |
| *12.3 Aspirate details* | pH value: | Colour of aspirate: | |
|  | pH test result time, 24-hr time format (HH:MM): | | |
|  | Remarks: | | |
| *12.4 X-ray details* | Type of X-ray performed: | 🞎 CXR  🞎 AXR | |
|  | The time when doctor confirmed the NGT location, 24-hr format (HH:MM):  (Reference to Patient Notes) | | |
|  | Location of NGT: | 🞎 Stomach  🞎 Oesophagus  🞎 Tracheobronchial tree  🞎 Others (please specify: ________________) | |
|  | Need for additional X-ray: | 🞎 Yes 🞎 No  If **Yes**, please complete the following: | |
|  |  | | |
|  | Repeated X-ray:  Type of X-ray performed: | 🞎 AXR  🞎 CXR | |
|  | The time when doctor confirmed the NGT location, 24-hr format (HH:MM):  (Reference to Patient Notes) | | |
|  | Location of NGT: | 🞎 Stomach  🞎 Oesophagus  🞎 Tracheobronchial tract  🞎 Others (please specify: _________________) | |
|  | Remarks: | | |

13. Complications or adverse events during NGT insertion

| 🞎 Repeated vigorous cough  🞎 Cyanosis  🞎 Nasal bleeding  🞎 Others: _____________________________________________________________  Action(s):  🞎 Withdraw NGT and re-insert  🞎 Withdraw NGT and fail to re-insert  🞎 Others, please specify ___________________________________________________________________  ___________________________________________________________________ ___________________________________________________________________ |
| --- |

14. Complications or adverse events occur during the capnography measurement

**_____________________________________________________________________________**

**_____________________________________________________________________________**

15. Others:

| **Cost of the reference test(s) used: (if applicable)**  Cost of X-ray: _________ (HK$)   - Unit Cost: HK$ 201   No. of X-rays performed: __________________(DN)    Cost of pH strips: _____ (HK$)   - Unit Cost: HK$ 0.45   No. of pH test strips consumed: _________________(DN)  **Cost of index test: _____________ (HK$)**  Cost of capnography tubing: ____(HK$)   - Unit Cost: HK$ 99   No. of tubing consumed: ________________(DN)  Cost of adaptive device between the NGT & capnography: ____(HK$)   - Unit Cost: HK$ 30   No of adaptive device consumed: ______________(DN)  X-ray procedural time* (HH:MM): __________________(RA)  *Starting time from the order of X-ray by a doctor, to the ending time when a doctor confirms the NGT location (as documented on the patient note). |
| --- |

**~ END ~**
